# Peripheral vascular complications following totally endoscopic cardiac surgery

**DOI:** 10.1186/s13019-021-01417-x

**Published:** 2021-03-20

**Authors:** Ling-chen Huang, Qi-chen Xu, Dao-zhong Chen, Xiao-fu Dai, Liang-wan Chen

**Affiliations:** 1 Department of Cardiovascular Surgery, Union Hospital, Fujian Medical University, Fuzhou, 350001 China; 2 Fujian Key Laboratory of Cardio-thoracic Surgery (Fujian Medical University), Fuzhou, China

**Keywords:** Totally endoscopic, Cardiac surgery, Vascular complication

## Abstract

**Background:**

Clinical application of minimally invasive cardiac surgery has increased annually. Cardiopulmonary bypass is established by peripheral cannulation during minimally invasive cardiac surgery. The methodology of peripheral cannulation has unique characteristics, which have associated risks and complications. Few studies have been conducted on this topic. In this study, we focused on complications of peripheral cannulation in totally endoscopic cardiac surgery.

**Methods:**

Patients who underwent totally endoscopic cardiac surgery with cardiopulmonary bypass established by peripheral cannulation at our institution between January 2019 and June 2020 were reviewed. Specific cannulation strategies and related cannulation complications were noted.

**Results:**

One hundred forty-eight patients underwent totally endoscopic cardiac surgery. One hundred forty-eight cannulations were performed in the femoral artery and vein, and eleven were performed in the internal jugular vein (combined with the femoral vein). The median size of the femoral artery cannula was 22Fr, and that of the venous canula was 24Fr. One patient died of retroperitoneal haematoma due to femoral artery injury. Three patients had postoperative lower limb oedema. One patient had a postoperative diagnosis of femoral vein thrombosis.

**Conclusions:**

Different from cannulation in patients with aortic dissection and aneurysms, femoral artery cannulation is safe in totally endoscopic cardiac surgery. Venous cannulation is characterized by a large-bore venous cannula and a short period of use. There are few reports about complications of venous cannulation. The main complication in this study was mechanical injury, and the key to preventing this injury is meticulous manipulation during surgery.

## Introduction

In recent years, minimally invasive surgery has been rapidly adopted in the field of cardiothoracic surgery due to its advantages of reducing surgical trauma and promoting rapid recovery [1–3]. With the advent of surgical devices such as thoracoscopic instruments and the Da Vinci robotic surgical platform, four major surgical approaches to minimally invasive cardiac surgery (MICS) have been developed. These approaches include the following: lower hemisternotomy [4], direct-vision right minithoracotomy [5], endoscopic right minithoracotomy [6], and robotic-assisted right minithoracotomy [7]. All these approaches require novel cannulation and perfusion strategies. Due to the continuous improvement of surgical techniques and instruments, the number of MICS with cardiopulmonary bypass (CPB) via peripheral vascular cannulation is increasing. At present, the most commonly used approaches at our institution are totally endoscopic cardiac surgery with CPB established through a femoral artery-vein bypass, with occasional internal jugular vein combined with femoral vein cannulation. We conducted a review of the literature and found that few articles have specifically examined the complications of cannulation in totally endoscopic cardiac surgery. Complications involving arterial cannulation are occasionally mentioned in the literature for extracorporeal membrane oxygenation (ECMO) therapy and cardiac surgery via retrograde femoral artery perfusion [8–10]. However, there is no literature specifically focused on complications after venous cannulation in totally endoscopic cardiac surgery, so it is clinically relevant to conduct the present study. This study is a single-centre experience of totally endoscopic cardiac surgery, with analysis focused on complications related to peripheral cannulation. It is not a comparative study aimed at the complications of different cannulation sites but rather a specific look at a large cohort that received peripheral cannulation during MICS, with the purpose of determining the level of safety and the related complications.

## Materials and methods

### Patients selection

From January 2019 to June 2020, one hundred forty-eight patients underwent totally endoscopic cardiac surgery with CPB establishment through peripheral cannulation and thus constitute the study population. For all patients, the medical records, operative notes, and discharge summaries were reviewed in the electronic medical record system and picture archiving and communication system. We focused on the cannulation site, the type of surgery, and the complications associated with cannulation, as well as the overall clinical outcome.

Among patients who underwent totally endoscopic approaches, we excluded patients with contraindications to peripheral cannulation. These include peripheral vascular disease and aortic artery diseases, suspected right pleural adhesions (including a history of prior right thoracic surgery) and the need for additional aortic valve surgery and/or coronary artery bypass grafting.

This article retrospectively reviews the early outcomes of complications associated with cannulation in totally endoscopic cardiac surgery. The study was reviewed and approved by the Institutional Review Committee of Union Hospital **(approval ID: No. 2020KY090)**, and the requirement for informed consent was waived because the study was retrospective.

### Surgical technique

**Fig. 1:**
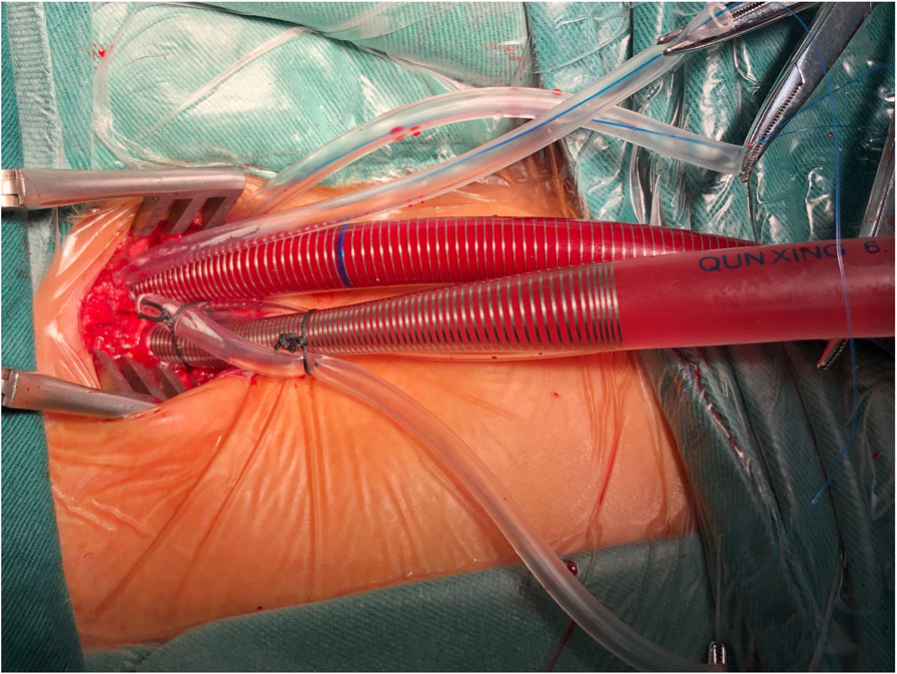
Femoral artery and vein cannulation performed with the Seldinger technique, without vessel clamping

We routinely perform totally endoscopic cardiac surgery with CPB establishment through femoral artery-vein bypass. We perform femoral artery and vein cannulation using open Seldinger-guided technique. Following the surgical exposure of the femoral artery and vein, the femoral vessels are then cannulated by guidewire inside a purse-string without vascular incision. In addition to being easier and faster to perform and not requiring an arteriotomy, this technique has the added benefit of continued perfusion of the distal limb, as no arterial snare is required. After general anaesthesia and skin disinfection, a groin incision approximately 2–4 cm long is made to expose the common femoral vessel. Two 5–0 Prolene sutures are used to place purse-string sutures on the femoral artery and vein. A transoesophageal echocardiography (TEE) probe is routinely placed to confirm guidewire placement when cannulation was performed. The guidewire is introduced into the femoral artery using the Seldinger technique and advanced towards the descending thoracic aorta. TEE is used to position and confirm the guidewire to ensure that the guidewire is within the aorta and to advance the cannula over the guidewire until the tip of the cannula is extended to the iliac artery. Venous cannulation is also established using the Seldinger technique, using a single two-stage venous cannula with the tip advanced to the level of the superior vena cava so that venous blood returning from the body can be drained. After CPB is established, TEE is used to confirm that the cannula is in the proper position and that a normal direction of blood flow is present. A Chitwood aortic cross-clamp is applied through the thoracic port, and cardioplegic arrest is obtained with antegrade delivery of a crystalloid solution. Gravity drainage is enhanced by a vacuum-assisted system. The cannulae in the femoral vein are removed at the end of surgery before neutralizing heparin is administered, and then the arterial cannula is removed (Fig. 1).

### Statistical analysis

Statistical analyses were conducted using SPSS 22.0. Quantitative data with a normal distribution are presented as the mean ± standard distribution; for nonnormally distributed data, the median is used.

## Results

**Fig. 2:**
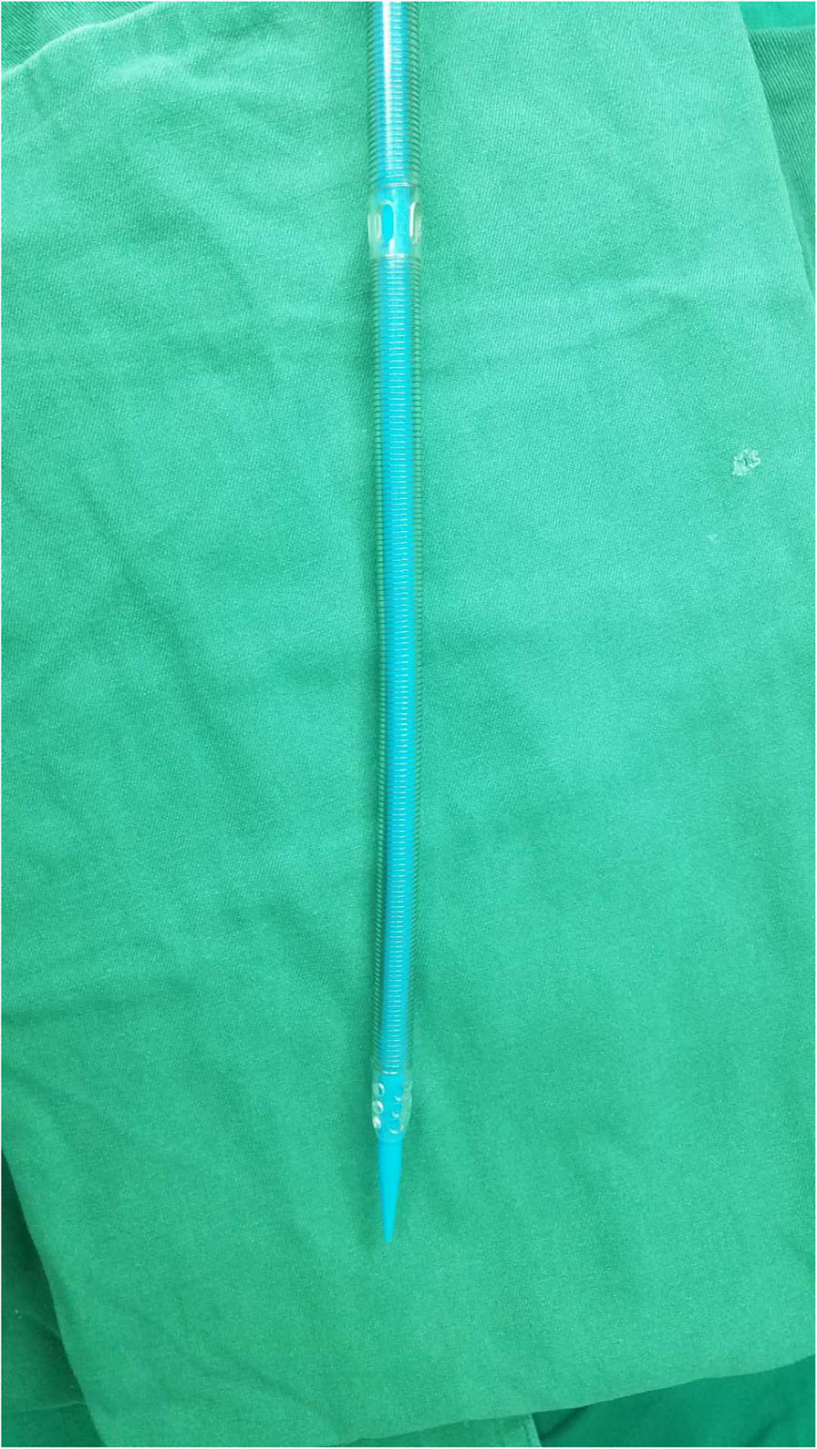
A single two-stage venous cannula

One hundred forty-eight patients underwent total endoscopic cardiac surgery. The mean age was 52.64 ± 12.13 years; 79 were female. The mean body mass was 22.76 ± 1.59 kg/m^2^. The median size of the femoral artery cannula was 22Fr, and the median venous cannula size [femoral venous cannulae, Kangxin Medical Instruments Co. Ltd.] (Fig. 2) was 24Fr.

Of 148 patients, one hundred and twenty-three patients (83.11%) underwent mitral valve surgery, sixteen patients (10.81%) underwent isolated atrial septal defect repair, and nine (6.08%) underwent isolated tricuspid valve surgery. Eighty-one patients (34.15% of 123 patients) underwent mitral valve replacement, and valvuloplasty was performed in forty-two patients (65.85%). Of the patients who underwent mitral valve surgery, thirty-six underwent concomitant tricuspid valvuloplasty (25 underwent DeVega procedures, and 11 underwent annuloplasty rings), six underwent atrial fibrillation ablation, and two underwent atrial septal repair. Two patients underwent median sternotomy due to severe indivisible plural adhesion and bleeding. The mean CPB time was 147.1 ± 38.7 min, and the aortic cross-clamp time was 101.6 ± 21.3 min. Of these procedures, isolated atrial septal defect repair and tricuspid valvuloplasty were performed in beating hearts. The demographic and Intra-operative data of the patients are shown in Table 1.

**Table 1:** Demographic and Intra-operative data

Item	Data
Male/Female	69/79
Age (years)	52.64 ± 12.13
BMI (kg/m^2^)	22.76 ± 1.59
Femoral artery cannula size (median)	22Fr
Femoral vein cannula size (median)	24Fr
Types of surgery	
Mitral valve surgery (n, %)	123 (83.11%)
Atrial septal defect repair (n, %)	16 (10.81%)
Tricuspid valve surgeries (n, %)	9 (6.08%)
Mitral valve surgery strategy	
Mitral valve repair (n, %)	81 (65.85%)
Mitral valve replacement (n, %)	42 (34.15%)
Concomitant procedures during MVS	
Tricuspid valve plasty	36 (24.32%)
Atrial fibrillation ablation	6 (4.05%)
Atrial Septal repair	2 (1.35%)
Convert to sternotomy (n, %)	2 (1.35%)
Cardiopulmonary bypass time (min)	147.1 ± 38.7
Aortic cross-clamping time (min)	101.6 ± 21.3

The post-operative data of the patients are detailed in Table 2. One elderly female patient without atherosclerosis risk factors such as smoking, hyperlipidaemia and hypertension died of multiple organ failure in the intensive care unit after surgery. The guide wire was unexpectedly withdrawn with US-guided access to the femoral vessel during the operation, and the arterial cannula injured the posterior wall of the femoral artery. Abdominal distension was found during CPB, and an exploratory laparotomy revealed retroperitoneal haematoma due to perforation of the femoral artery.

**Table 2:** Postoperative Data

Item	TA group
Early Mortality (<30 days) (n, %)	1 (0.68%)
Morbidity of Artery Cannulation	
Artery injury (n, %)	1 (0.68%)
Neurological complications	0
Embolism	0
Lower limb ischemia	0
Infection	0
Morbidity of Venous Cannulation	
Lower limb edema (n, %)	3 (2.03%)
Thrombosis (n, %)	1 (0.68%)
Hematoma formation	0
Venous injury during surgery (n, %)	9 (6.08%)
Infection	0

**Fig. 3:**
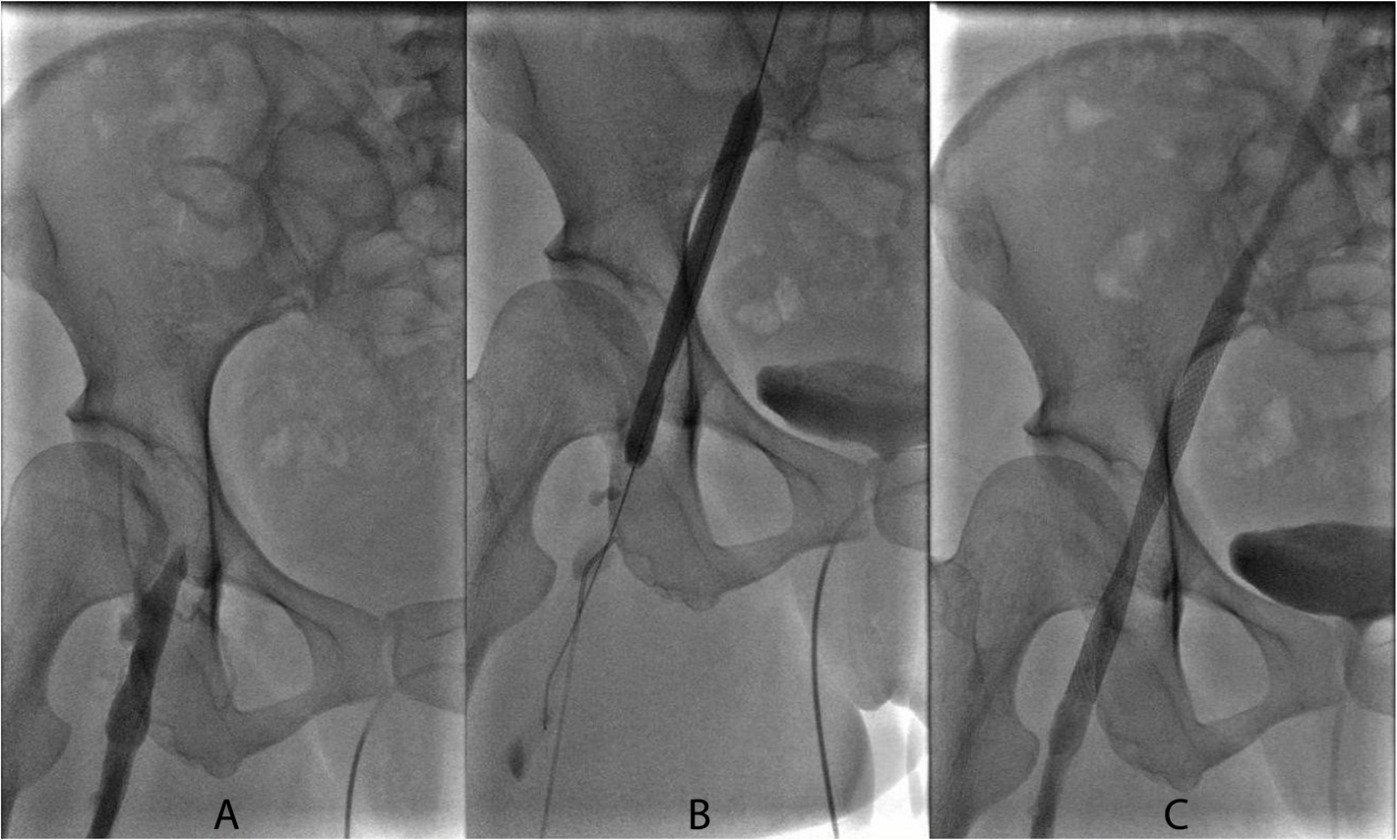
**a** A case of venous complication: angiographic showed the thrombosis in right iliofemoral vein. **b** The vein was dilated using an angioplasty balloon catheter. **c** Unobstructed of the right iliofemoral vein after covered stent implantation

Postoperative lower limb oedema occurred in a total of three patients, including one patient without remission during the follow-up period. Another patient was an older female. The patient was obesity. Compared the femoral vein visually with our venous cannula, the diameter of the femoral vein and the cannula was similar, and it was difficult to apply single two-stage femoral venous cannulae, causing vessel injury. In addition, after removal of the femoral vein cannulae, the femoral vein had to be repaired due to bleeding. The patient was bedridden postoperatively. This patient had a postoperative diagnosis of femoral vein thrombosis. Immediately after her diagnosis, she was transferred to the vascular surgery department for catheter-directed thrombolysis combined with stent placement for acute femoral vein thrombosis, and warfarin anticoagulation was continued (Fig. 3).

## Discussion

MICS is a landmark event in the history of cardiology; it minimizes many of the side effects associated with median sternotomy and extracorporeal circulation and has now become a widely adopted method [11, 12]. MICS is technically unique from conventional cardiac surgery and requires the use of novel perfusion strategies. The cannulation technique is an essential element of MICS, and there are multiple cannulation strategies for each type of MICS, including routine use of vacuum-assisted venous drainage, alternate arterial and venous cannulation sites, and special cannulae designed for minimally invasive procedures. In this article, we focus on the vascular complications and related preventive measures associated with totally endoscopic cardiac surgery with CPB established by peripheral cannulation.

The vascular complications of totally endoscopic cardiac surgery include leg ischaemia, arterial injury, arterial dissection, pseudoaneurysm, embolism, venous thrombosis and arteriovenous fistula. Few studies have focused on the incidence of vascular complications associated with peripheral cannulation in MICS. Because most of these complications are relatively easy to manage and there are few long-term complications, the exact incidence is not yet clear.

Greelish and colleagues reported a 3.9% incidence of femoral artery injury during femoral artery cannulation [13]. In our study, all patients with femoral artery cannulation were treated using the modified Seldinger technique. Relevant research suggests that femoral artery cannulation offers a safe alternative to aortic cannulation for CPB and is especially important in MICS [14]. The biggest issue with femoral artery cannulation is that many people question the risk of increased mortality, neurological complications and embolism [15, 16]. Different from cannulation in patients with aneurysms and aortic dissection, which involve thrombi in the false lumen and thus are associated with a high rate of mortality, stroke and other complications, including retrograde cerebral embolization [17, 18]. We point out that the literature on femoral artery cannulation for MICS is rather scant and represents only the experience of a single centre. Cannulation in MICS did not lead to this risk in our study, and there were no neurological complications or arterial embolism events in this group of patients. There were no complications related to retrograde perfusion. Only one patient (an elderly woman who did not smoke or have other risk factors) died of retroperitoneal haematoma due to femoral artery injury. Some published articles indicated that a retrograde perfusion strategy for MICS increases the neurologic risk, especially in elderly patients [15, 16, 19]. Grossi recommended that preoperative evaluation of the descending aorta and distal arteries be required when femoral artery perfusion is performed in older patients [19]. Preoperative computed tomographic angiography (CTA) of the descending aorta and femoral artery may be warranted in elderly patients. However, there was only one arterial vascular complication in our study, which could be considered as a low complication rate. Angiography may minimize the risk of neurological complications and embolism, but there are also risks such as allergies to contrast agents, renal failure and increased costs that should be considered in combination with the pros and cons.

In our institution we usually perform vascular ultrasound of lower extremity in obese or elderly patients before surgery. We occasionally request a pre-operative CT scan in order to assess the aorta and femoral vessels in some cases where we think it is necessary. Besides, the surgeon, anaesthesiologist and perfusionist consult preoperatively, and if there is a consensus to re-evaluate the femoral vasculature preoperatively, the anaesthesiologist will use an ultrasound probe to re-assess the lower extremity vasculature of the patient.

The diameter of the femoral artery in the Chinese population is generally sufficient to place an arterial cannula of 20-22Fr, and since the Chinese population is less likely to be obese, 22Fr is sufficient for the majority of people. If the measured diameter of the femoral vein is not adequate, we use a combination of jugular vein and femoral vein cannulation instead of a single two-stage cannula.

Lower limb ischaemia is also a potential risk factor for femoral artery cannulation. Body mass index, cannula size and operation duration can be considered risk factors for leg ischaemia in peripheral cannulation during MICS [20]. For minimally invasive endoscopic cardiac surgery, the duration of surgery and the duration of CPB are usually shorter, and there is no circulatory arrest. Moreover, small femoral arteries can also be cannulated with a Dacron side graft or bilateral cannulation and even axillary artery cannulation to prevent artery-related complications. At this time, there are alternative methods, such as arterial perfusion cannulae with angled side holes [21]. The appeal method is also not used in practice. In this study, the rate of artery-related complications was only 0.68%, and lower limb ischaemia never occurred.

Although the number of patients included in the current study is small, the results are still of clinical significance and indicate femoral artery cannulation is a safe technique in totally endoscopic cardiac surgery.

In terms of complications of femoral venous cannulation, unlike ECMO, aortic dissection and central venous catheterization, totally thoracoscopic surgery is characterized by the addition of femoral venous cannulae, a shorter indwelling time and a larger conduit diameter due to the large flow requirement. Few studies have been conducted on total endoscopic femoral venous cannulation, no specific studies have been retrieved, and only a few studies have been conducted on central venous catheterization [22, 23]. For this kind of central venous catheter, with a long indwelling time and small diameter, there is a high risk of infection and thrombosis. The main mechanical complications were haematoma formation and arterial puncture injury. However, for vena cava cannulation, due to the large venous cannula diameter, mechanical complications often manifest as vessel injury. Venous injuries were relatively frequent in our study. Prevention methods include estimating the femoral vein diameter and perform internal jugular vein cannulation preoperatively and placing a right-angle superior vena cava cannula during the operation. In our study, intraoperative CPB flow met the basic needs, and additional internal jugular vein and superior vena cava cannulation was rarely required; however, there were many cases of femoral vein repair during the operation.

The most common symptoms were postoperative lower limb oedema and colour ultrasound indicated a reduction of blood flow. With the improvement of the suture and cannulation technique (the shape of the purse-string suture should be an oblate ellipsoid, with the long axis perpendicular to the long axis of the femoral vessel), this incidence of this complication was gradually reduced. The symptoms disappeared in most patients during follow-up. This may be related to the formation of lateral branches. Only one patient had persistent femoral vein stenosis. Postoperative thromboembolism occurred in one patient who, according to the theory of the three elements of Virchow thrombosis, was at high risk for femoral vein thrombosis. Therefore, for this kind of patient, we need to pay careful attention during the operation and avoid long-term postoperative bedrest after surgery.

Based on the results of our literature review [24, 25], we included studies related to peripheral cannulation complications, including cannulation complications of ECMO, aneurysm and aortic dissection surgery, and minimally invasive cardiac surgery, in Table 3. We found that cannulation complications in ECMO, MICS and thoracic aortic surgery each have their own characteristics, with different incidence rates and inconsistent research reporting. More studies have been performed on ECMO and lower extremity ischaemia and deep vein thrombosis (DVT) complications due to the long-term indwelling of the cannula; thoracic aortic surgery is more concerned with mortality and neurological complications due to the presence of false lumen thrombosis or atherosclerotic plaque. Mortality and neurological complications of thoracic aortic surgery are of greater concern due to the presence of false lumen thrombosis and atherosclerotic plaque. Examining the MICS data, we found that femoral artery cannulation has unique features, such as not being associated with as many cerebral complications as aortic dissection and aneurysm surgery and not having as high an incidence of lower extremity ischaemia because of the short duration of use. We also found that there are no studies related to complications of venous cannulation in thoracoscopic surgery. Therefore, our study has obvious clinical relevance.

**Table 3:** Eligible studies on complications of peripheral cannulation (Perioperative data)

Author, date and topic	Patient characteristics	Outcome	Result	Comments
Yau [8] 2019 Ipsilateral limb ischaemia in femoral cannulation ECMO	154 patients from March 2011 to September 2016 underwent femoral artery cannulation (*n* = 154)	Mortality Leg ischaemia	59.7% 22%	Femoral cannulation ECMO associated with limb ischaemia
Greelish [13] 2003 Minimally invasive mitral valve repair	358 patients from August 1996 to May 2002 underwent femoral artery cannulation (*n* = 128) femoral vein cannulation (*n* = 224)	Mortality Stroke Aortic dissection Artery injury DVT	0% 1.9% 1.5% 1.4% 0.8%	Ten arterial and three venous complications
Saadat [14] 2016 Minimally invasive cardiac surgery	346 patients from September 2012 to September 2013 underwent femoral artery cannulation (*n* = 72)	Mortality Stroke	6.94% 1.39%	Focus on the safety of femoral cannulation in MICS
Murzi [15] 2017 Minimally invasive cardiac surgery	1632 patients from 2003 to 2015 underwent femoral artery cannulation (*n* = 141)	Mortality Stroke Artery injury	0.7% 3.5% 1.4%	Retrograde perfusion associated with higher incidence of stroke in MICS
Bedeir [16] 2015 Minimally invasive cardiac surgery	384 patients from January 2004 to June 2012 underwent femoral artery cannulation (*n* = 57)	Mortality Stroke	0% 5.3%	Retrograde perfusion associated with higher incidence of stroke in MICS
Tarui [20] 2018 Minimally invasive cardiac surgery	162 patients from May 2014 to December 2016 underwent femoral artery cannulation (*n* = 162)	Stroke Aortic dissection Artery injury	0% 0% 0%	Analysis of risk factors for leg ischaemia in peripheral cannulation MICS
Marasco [21] 2018 Novel bidirectional perfusion cannula	15 patients from August 2016 to May 2017 underwent femoral artery cannulation (n = 15)	Mortality Aortic dissection Artery injury Leg ischaemia	0% 0% 0% 0%	Novel bidirectional perfusion cannula can provide stable distal perfusion
Bisdas [24] 2011 Vascular complications in femoral cannulation for ECMO	174 patients from January 2005 to December 2009 underwent venoarterial mode ECMO (*n* = 143) or venovenous mode ECMO (*n* = 31)	-Mortality -Vascular complications	61% 10%	Vascular complications after ECMO support are not associated with higher mortality rates
Kamiya [25] 2009 The site of cannulation for repair of ascending aortic dissection	242 patients from January 1988 to September 2007 underwent: ascending aorta cannulation(*n* = 82) femoral artery cannulation(*n* = 153)	Mortality Stroke	23% 4.5%	The cannulation site should be chosen according to the patient’s pathology and status

The small sample size is a limitation of this study, and a larger number of surgical cases would have allowed for stronger conclusions to be drawn. Other limitations include the nature of the retrospective analysis, the single-centre experience, and the lack of matched controls for patients with different cannulation techniques during the study period. However, despite these limitations, our findings provide new evidence on the complication rates of peripheral cannulation in MICS.

## Conclusion

Overall, endoscopic cardiac surgery is associated with a low incidence of vascular complications. The risk of associated complications in femoral artery cannulation is low, and cannulation can be safely performed. Femoral vein cannulae in endoscopic cardiac surgery are characterized by large cannula diameters and short durations of use, and there are few relevant studies. There is a certain incidence of complications related to femoral venous cannulation in clinical practice that should be considered. We believe that preventing mechanical injuries during surgery is key. Peripheral vascular complications following totally endoscopic cardiac surgery can be safely treated but are nonnegligible.

## Data Availability

Data sharing not applicable to this article as no data sets were generated or analyzed during the current study.

## Abbreviations

CPB: Cardiopulmonary bypass
ECMO: Extracorporeal Membrane Oxygenation
MICS: Minimally invasive cardiac surgery
TEE: Transesophageal echocardiography
CTA: Computed tomographic angiography
DVT: Deep vein thrombosis

## References

[1] Cohn LH, Adams DH, Couper GS, Bichell DP, Rosborough DM, Sears SP, Aranki SF (1997). Minimally invasive cardiac valve surgery improves patient satisfaction while reducing costs of cardiac valve replacement and repair. Ann Surg.

[2] Casselman FP, van Slycke S, Wellens F (2003). Mitral valve surgery can now routinely be performed endoscopically. Circulation..

[3] Ritwick B, Chaudhuri K, Crouch G, Edwards JR, Worthington M, Stuklis RG (2013). Minimally invasive mitral valve procedures: the current state. Minim Invasive Surg.

[4] McClure RS, Cohn LH, Wiegerinck E (2009). Early and late outcomes in minimally invasive mitral valve repair: an eleven-year experience in 707 patients. J Thorac Cardiovasc Surg.

[5] Ward AF, Grossi EA, Galloway AC (2013). Minimally invasive mitral surgery through right mini-thoracotomy under direct vision. J Thorac Dis.

[6] Vanermen H, Farhat F, Wellens F, Geest R, Degrieck I, Praet F, Vermeulon Y (2000). Minimally invasive video-assisted mitral valve surgery: from port-access towards a totally endoscopic procedure. J Card Surg.

[7] Carpentier A, Loulmet D, Carpentier A (1996). First open heart operation (mitral valvuloplasty) under videosurgery through a minithoracotomy. Comptes Rendus de l’Academie des Sciences III.

[8] Yau P, Xia Y, Shariff S, Jakobleff WA, Forest S, Lipsitz EC, Scher LA, Garg K (2019). Factors associated with Ipsilateral limb ischemia in patients undergoing femoral Cannulation extracorporeal membrane oxygenation. Ann Vasc Surg.

[9] Ayyash B, Tranquilli M, Elefteriades JA (2011). Femoral artery cannulation for thoracic aortic surgery: safe under transesophageal echocardiographic control. J Thorac Cardiovasc Surg.

[10] Fusco DS, Shaw RK, Tranquilli M, Kopf GS, Elefteriades JA (2004). Femoral cannulation is safe for type a dissection repair. Ann Thorac Surg.

[11] Ryan WH, Brinkman WT, Dewey TM, Mack MJ, Prince SL, Herbert MA (2010). Mitral valve surgery: comparison of outcomes in matched sternotomy and port access groups. J Heart Valve Dis.

[12] Svensson LG, Atik FA, Cosgrove DM (2010). Minimally invasive versus conventional mitral valve surgery: a propensity-matched comparison. J Thorac Cardiovasc Surg.

[13] Greelish JP, Cohn LH, Leacche M, Mitchell M, Karavas A, Fox J, Byrne JG, Aranki SF, Couper GS (2003). Minimally invasive mitral valve repair suggests earlier operations for mitral valve disease. J Thorac Cardiovasc Surg.

[14] Saadat S, Schultheis M, Azzolini A, Romero J, Dombrovskiy V, Odroniec K, Scholz P, Lemaire A, Batsides G, Lee L (2016). Femoral cannulation: a safe vascular access option for cardiopulmonary bypass in minimally invasive cardiac surgery. Perfusion..

[15] Murzi M, Cerillo AG, Gasbarri T, Margaryan R, Kallushi E, Farneti P, Solinas M (2017). Antegrade and retrograde perfusion in minimally invasive mitral valve surgery with transthoracic aortic clamping: a single-institution experience with 1632 patients over 12 years. Interact Cardiovasc Thorac Surg.

[16] Bedeir K, Reardon M, Ramchandani M, Singh K, Ramlawi B (2015). Elevated stroke risk associated with femoral artery Cannulation during mitral valve surgery. Semin Thorac Cardiovasc Surg.

[17] Ohno N, Minatoya K (2020). sArterial cannulation to establish cardiopulmonary bypass during surgery for acute aortic dissection. Surg Today.

[18] Tiwari KK, Murzi M, Bevilacqua S, Glauber M (2010). Which cannulation (ascending aortic cannulation or peripheral arterial cannulation) is better for acute type a aortic dissection surgery?. Interact Cardiovasc Thorac Surg.

[19] Grossi EA, Loulmet DF, Schwartz CF, Ursomanno P, Zias EA, Dellis SL, Galloway AC (2012). Evolution of operative techniques and perfusion strategies for minimally invasive mitral valve repair. J Thorac Cardiovasc Surg.

[20] Tarui T, Miyata K, Shigematsu S, Watanabe G (2018). Risk factors to predict leg ischemia in patients undergoing single femoral artery cannulation in minimally invasive cardiac surgery. Perfusion..

[21] Marasco SF, Tutungi E, Vallance SA, Udy AA, Negri JC, Zimmet AD, McGiffin DC, Pellegrino VA, Moshinsky RA (2018). A phase 1 study of a novel bidirectional perfusion cannula in patients undergoing femoral Cannulation for cardiac surgery. Innovations (Phila).

[22] McGee DC, Gould MK (2003). Preventing complications of central venous catheterization. N Engl J Med.

[23] Parienti JJ, Mongardon N, Mégarbane B, Mira JP, Kalfon P, Gros A, Marqué S, Thuong M, Pottier V, Ramakers M, Savary B, Seguin A, Valette X, Terzi N, Sauneuf B, Cattoir V, Mermel LA, du Cheyron D (2015). Intravascular complications of central venous catheterization by insertion site. N Engl J Med.

[24] Bisdas T, Beutel G, Warnecke G, Hoeper MM, Kuehn C, Haverich A, Teebken OE (2011). Vascular complications in patients undergoing femoral cannulation for extracorporeal membrane oxygenation support. Ann Thorac Surg.

[25] Kamiya H, Kallenbach K, Halmer D, Ozsoz M, Ilg K, Lichtenberg A, Karck M (2009). Comparison of ascending aorta versus femoral artery cannulation for acute aortic dissection type a. Circulation..

